# Recommendations and exploration of diagnosis and treatment of critical and refractory immune checkpoint inhibitor‐associated adverse events

**DOI:** 10.1111/1759-7714.13553

**Published:** 2020-07-06

**Authors:** Peng Song, Hanping Wang, Xiaoyan Si, Xiaoxiao Guo, Yue Li, Jiaixin Zhou, Lian Duan, Li Zhang, Mengzhao Wang

**Affiliations:** ^1^ Department of Respiratory Medicine Peking Union Medical College Hospital, Chinese Academy of Medical Science & Peking Union Medical College Beijing China; ^2^ Department of Cardiology Peking Union Medical College Hospital, Chinese Academy of Medical Science & Peking Union Medical College Beijing China; ^3^ Department of Gastroenterology Peking Union Medical College Hospital, Chinese Academy of Medical Science & Peking Union Medical College Beijing China; ^4^ Department of Rheumatology Peking Union Medical College Hospital, Chinese Academy of Medical Science & Peking Union Medical College Beijing China; ^5^ Department of Endocrinology Peking Union Medical College Hospital, Chinese Academy of Medical Science & Peking Union Medical College Beijing China

**Keywords:** Adverse events, critical, refractory, immune checkpoint inhibitor

## Abstract

The application of immune checkpoint inhibitors (ICIs) has rewritten many malignant tumor treatment strategies and become another milestone in tumor treatment. This article summarizes the latest domestic and international guidelines and consensus regarding the diagnosis and treatment of grade 3–4 immune‐related adverse effects (irAEs). Included are the findings of annual meetings of the European Society for Medical Oncology (ESMO), National Comprehensive Cancer Network/American Society for Clinical Oncology (NCCN/ASCO), the Society for Immunotherapy of Cancer (SITC), and the Chinese Society of Clinical Oncology (CSCO) with review of case reports and related reviews of irAEs that were published before 20 May 2019. The recommendations for the diagnosis and treatment of irAEs are supplemented, highlighting the successful application of specific immunosuppressive drugs in different irAEs, including IL‐6 blockade, anti‐CD20 monoclonal antibody, antitumor necrosis factor alpha and anti‐integrin 4 monoclonal antibodies, thrombopoietin receptor agonist, and antithymocyte globulin. This article questions the use of steroid hormones for irAEs in ultra‐large doses, upgrades, and repeated use, and emphasizes that it is important to note secondary infections, tumor progression, and the inability to meet the challenges of ICIs. Herein, we propose the principle of “stepping down treatment” for critical and refractory irAEs, and suggest that the use of specific immunosuppressive drugs such as cytokine‐targeted drugs should be initiated as soon as possible. Many irAEs in the era of immunotherapy are unprecedented in the era of traditional chemotherapy and small molecule targeted therapy, and this constantly challenges the knowledge reserve and clinical skills of oncologists. Therefore, the establishment of a multidisciplinary discussion system for cancer is extremely important.

## Abstract 2

Many irAEs in the era of immunotherapy are unprecedented in the era of traditional chemotherapy and small molecule targeted therapy, and this constantly challenges the knowledge reserve and clinical skills of oncologists. Therefore, the establishment of a multidisciplinary discussion system for cancer is extremely important.

## Introduction

The application of Coley's toxin in 1863 was one of the first immunotherapy treatments for tumors. Other immunotherapy treatments consist of Bacillus Calmette‐Guerin (BCG), interferon‐α (IFN‐α), interleukin‐2 (IL‐2), major histocompatibility complex (MHC), and tumor necrosis factor (TNF).^1^, ^2^ Recently, immune checkpoint inhibitors (ICIs) have also been employed. Unlike traditional chemotherapy and targeted therapy, ICIs do not directly kill tumor cells, but rather, they target immune cells to enhance the antitumor immune response and bring long‐term clinical benefits to patients with advanced tumors. Examples are programmed cell death protein 1 (PD‐1)/programmed cell death protein ligand 1 (PD‐L1) inhibitor and cytotoxic T lymphocyte‐associated antigen 4 (CTLA‐4) inhibitor. However, while PD‐1/PD‐L1 and CTLA‐4 inhibitors provide long‐term and sustained clinical benefits to patients with advanced tumors, they may also cause systemic immune‐related adverse effects (irAEs) that can be toxic and life‐threatening.

Mild irAEs (grade 1–2) and most grade 3–4 irAEs can be well controlled after early steroid treatment. Some patients can receive ICI treatment again, but there are still a small number of irAEs with severe clinical manifestations. The critical or refractory type can be effectively controlled by steroids. However, ICIs should be discontinued if the patient subsequently undergoes life‐threatening, uncontrollable irAEs, adverse reactions secondary to steroid use, or primary tumor progression.

There are guidelines that provide detailed recommendations for the management of common irAEs, and also emphasize early recognition and management, as well as differential diagnosis (for infection and other complications, tumor progression, and the presence and activity status of underlying diseases), However, there is less guidance available for critical and refractory irAEs, and what is available is often only of a general nature, or the clinically existing “refractory irAEs” are not described as a unique type of irAE.

Clinically, the most optimal course of action is to increase the success rate of irAE management by overcoming refractory irAEs. The treatment for severe irAEs (grade 3 to 4) in the major guidelines are currently similar, that is, to follow the principles of CTCAE‐4.03.^3^ Patients with grade 3–4 irAEs should be hospitalized and receive any necessary intensive care unit (ICU) treatment. Patients whose symptoms have not been relieved after three to five days of systemic steroid treatment can be further treated under the guidance of a specialist. ICIs should be temporarily or permanently discontinued, and if grade 4 toxicity is noted, the use of ICIs should be permanently stopped. For systemic steroid treatment, it is recommended to use intravenous methylprednisolone 1–2 mg/kg/day for three consecutive days.

If symptom relief occurs, the dose should gradually be reduced to 1 mg/kg/day for four to six weeks. However, the guidelines provide no additional recommendations for the specific types, dosages, and dosage forms of steroid hormones. There are no profound descriptions of the adverse reactions secondary to hormones, and there are no further recommendations for the subsequent treatment of hormone‐insensitive persons. Therefore, these critical and refractory irAEs are still the main clinical problems. This article summarizes the progress of clinical research for treatment of critical and refractory irAEs in recent years and solution strategies, and aims to provide a reference to assist oncology researchers in solving problems.

## Methods and basis

This article summarizes the existing guidelines, consensus, and literature, including: (i) A summary of the recommendations for grade 3–4 toxicity from five guidelines and consensus, as long as one of the guidelines and consensus provides recommendations. Additionally, differences and inconsistencies in the consensus of the various guidelines under special circumstances are noted. (ii) PubMed and Embase databases were searched (as of 20 May 2019), with retrieval of nivolumab, pembrolizumab, atezolizumab, avelumab, durvalumab, ipilimumab, tremelimumab, or joint use; mediated AEs or immune‐mediated toxicity; English; screening case). A total of 232 cases were reviewed and summarized one by one, and the relevant review articles and literature were reviewed, supplementing the type names of irAEs not mentioned in the guidelines and consensus. In particular, the guidelines supplement treatment of the critical irAEs. (iii) Several types of immunosuppressive drugs that may be related to the treatment of irAEs are recommended.

## Recommendation summary and description of each system in the guide and consensus and the suggestions in this article

Following the introduction of guidelines for irAEs, the Chinese Society of Clinical Oncology CSCO is divided into two parts: consensus recommendations and other recommendations. It mainly introduces the names and treatment strategies for critical irAEs, and notes the types of irAEs and recommendations that are not mentioned in the current guidelines and consensus.

### Skin rash system

According to existing guidelines and consensus, maculopapular rash, itching, and reactive skin capillary hyperplasia (CCEP) are divided into three grades, with no grade 4 toxicity, and refractory AEs are not considered. CCEP is only seen in the CSCO guide. Severe rashes with grade 3–4 toxicity are defined as bullous dermatitis, Stevens‐Johnson syndrome (SJS)/toxic epidermal necrolysis (TEN), associated eosinophilia, and drug rash with eosinophilia and systemic symptoms (DRESS), and these can simultaneously occur.

Treatment strategies include permanent discontinuation of ICIs and prednisone/methylprednisolone 1 mg/kg/day–2 mg/kg/day, requiring hospitalization. Skin changes such as psoriasis and vasculitis with purpura can be critical and refractory^4^ and treated with prednisone. Studies and case reports have shown that azathioprine, mycophenolate mofetil, methotrexate, tetracycline antibiotics (tetracycline, doxycycline, and minocycline), dapzon, and nicotinamide can be used as effective steroid replacement drugs. A retrospective study of 21 patients with psoriasis noted a high incidence of ICI‐induced psoriasis in men 18/21 (85.7%). Of the 21 patients, two psoriasis patients (9.5%) received systemic hormone therapy, one (4.8%) patient received etrenic acid treatment, and one patient (4.8%) received phototherapy; of these, remission after treatment occurred for 19 cases (90.5%). For psoriasis in the acute phase, treatment with anti‐TNFα, IL‐1 blockers, and anti‐IL‐23 and ‐12 monoclonal antibodies may be considered. Steroid hormones can be used to treat severe psoriasis that includes joint pain. IL‐1 blockers, and anti‐IL‐12 and ‐23 monoclonal antibodies have been successfully used for refractory psoriasis that is insensitive to anti‐TNFα. Anti‐CD20 monoclonal antibody is recommended for the treatment of dermatitis.^5^

### Endocrine system

The endocrine system toxicity described in the guidelines/consensus includes inflammation of the thyroid, adrenal gland, pituitary, and pancreas, and hyperglycemia can be caused by pancreatic involvement. The use of steroid hormones is not recommended, and grade 4 toxicity does not involve discontinuation immunotherapy. Hormone replacement and symptomatic treatment can again challenge ICIs after recovery to 1 degree. Patients with pancreatic islet injury cannot fully recover their pancreatic islet function regardless of whether they are treated with hormones or not. Patients will need to use insulin throughout their lives to control blood glucose. If gonad, thyroid, and adrenal function cannot be restored, life‐long hormone replacement therapy is required. Ipilimumab treatment should be used for pituitary inflammation. For these injuries of the endocrine system, the current guidelines/consensus only recommend supplementing the corresponding hormone. In addition, there is currently no corresponding treatment opinion for patients with mumps and salivitis.

### Digestive system

Digestive system toxicity includes liver toxicity, pancreatic toxicity (acute pancreatitis), gastrointestinal toxicity (diarrhea/colitis), esophagitis, gastritis, duodenitis, enteritis, cholangitis, and pancreatitis.

#### Liver toxicity

Each guideline/consensus lists different grades for liver toxicity. The European Society for Medical Oncology (ESMO) and National Comprehensive Cancer Network/American Society for Clinical Oncology (NCCN/ASCO) guidelines do not include bilirubin elevation in the diagnostic grade. The Society for Immunotherapy of Cancer (SITC) grade 4 toxicity standard is low, whereas that for CSCO is relatively comprehensive. Only CSCO guidelines require that viral hepatitis (HBC and HCV) be confirmed if irAE treatment provides no benefits, and the prevalence of these two types of hepatitis are high in China. The general treatment principle is the use of steroid hormones, mycophenolate mofetil, and tacrolimus. When the curative effect is not good, unique treatment is recommended. Antithymocyte globulin (ATG) is the recommended treatment when hormones are ineffective, and infliximab is not recommended because of its excessive risk. It is recommended that immunotherapy should be permanently discontinued when liver toxicity occurs. It is not advocated to switch to another agent in the same class of ICIs. Switching to a different type of ICI should be carefully considered, eg, the replacement of CTLA‐4 monoclonal antibody with PD‐1 and PD‐L1 monoclonal antibodies.

Changes in the ratio of aspartate aminotransferase (AST)/alanine aminotransferase (ALT) may reflect the presence of damage outside the liver. It should also be noted that bilirubin and liver enzymes do not simultaneously increase. Bile enzyme separation often indicates an extreme deterioration of liver function and life‐threatening emergencies, but whether it has the same clinical significance in the category of irAEs is unclear and requires additional exploration. In addition, there have been cases of successful application of IL‐6, anti‐CD20, and anti‐TNFα monoclonal antibodies for the treatment of critical and refractory hepatotoxicity, and the most optimal treatment has yet to be further explored.^5^

#### Pancreatic toxicity (acute pancreatitis)

Only the NCCN/ASCO guidelines list pancreatic toxicity as an independent toxic response, and their recommendation is to stop ICI and administer steroid therapy at level 2. Grade 3 to 4 pancreatic toxicity is defined as an increase in amylase or lipase to >3 times the upper limit of normal, and/or radiological abnormalities, and/or moderate abdominal pain or vomiting, hemodynamic instability, and changes in blood sugar.

The NCCN/ASCO guidelines recommend steroid therapy for pancreatic toxicity. If the effect is poor, it can be replaced with mycophenolate. Other guidelines do not mention this toxicity or treatment recommendations. Due to the small number of reported cases and insufficient treatment experience, there is no corresponding report or recommendation for treatments other than steroid hormones and mycophenolate, but from the perspective of the pathogenesis of irAEs, specific immunosuppressive treatment is worthy of further exploration.

#### Gastrointestinal toxicity

Gastrointestinal toxicity mainly manifests as diarrhea or colitis. According to the opinion of CSCO, steroid hormone treatment is recommended for grade 2 toxicity, and if it is ineffective, infliximab is recommended.

For grade 3–4 diarrhea, steroid hormones can be started without waiting for a colonoscopy. If hormones are not effective at 48 hours, consider adding infliximab; mycophenolate is rarely recommended. The ESMO guidelines do not exclude the use of antidiarrheal drugs. Other guidelines do not recommend antidiarrheal drugs.

For the first‐onset initial treatment, clinically typical grade 3–4 diarrhea, you can initiate steroid hormones without waiting for a colonoscopy. However, for refractory irAEs with long‐term use of steroid hormones, it is necessary to perform a colonoscopy as soon as possible to rule out infection. Secondary infections such as cytomegalovirus (CMV) and *Clostridium difficile*, and cases of long‐term repeated diarrhea may occur.

For refractory enteritis diarrhea, especially when the anti‐TNFα monoclonal antibody is not effective, IL‐1 and ‐6 inhibitors, and IL‐12, ‐17, and ‐23 monoclonal antibodies can be used.^5^ Surgical drainage and perforation repair should be performed when necessary.^6^ In addition, there are cases of gastrointestinal toxicity associated with esophagitis, gastritis, duodenitis, enteritis, cholangitis, and pancreatitis.

### Pulmonary toxicity

#### Interstitial lung injury

Acute interstitial pneumonia or diffuse alveolar damage syndrome (DADS) is a critical and life‐threatening event. Other lung diseases such as inflammatory pneumonia and sarcomatoid pulmonary granulomatosis are relatively mild, but are sometimes difficult to distinguish from infection or disease progression. For grade 3 pneumonia and above, hospitalization, empirical use of broad‐spectrum antibiotics, and high‐dose intravenous steroid therapy (such as (methyl)prednisolone 1–4 mg/kg/day or equivalent drug) are recommended. It should be noted that 10%–15% of patients may not be sensitive to hormone therapy. Thus, adding immunosuppressive therapy such as infliximab, mycophenolic acid, or cyclic phosphoramide to patients who have not improved on corticosteroid treatment after two days is recommended. These guidelines recommend that steroid hormones should be slowly reduced and then stopped over four to eight weeks. Intravenous immunoglobulin and ICU support are recommended. Bronchoscopy should guide treatment and become routine because it can correct deviations from empirical treatment.

Although the mortality rate from lung interstitial injury is high, in addition to irAEs being difficult to control, many patients die from uncontrolled infections. To avoid this, obtaining an in‐depth understanding of drugs such as cytokine antagonists might become a new research direction.^5^ There are also reports of Good‐pasture syndrome after ICI treatment, which should be identified as quickly as possible.^7^

#### Pulmonary sarcoidosis

Only the CSCO guidelines indicate that extrapulmonary (eye, myocardial, nervous system, and kidney) sarcoidosis and sarcoidosis accompanied by hypercalcemia are serious. The recommended dose of steroid hormones is 1–2 mg/kg, which are gradually discontinued within two to four weeks. The SITC guidelines indicate that such treatment is inappropriate. If clinical application of infliximab is considered, tuberculosis should be first excluded.

The pathological diagnosis of pulmonary nodules is essential and is easily confused with pseudoprogression and infection. Sarcoidosis‐like changes can occur not only in the lungs and lymph nodes, but also in the abdominal lymph nodes and brain parenchyma.^8^ New lesions that are inconsistent with the overall condition should be identified by tissue biopsy.

### Rheumatoid joint, bone, and muscle toxicity

#### Rheumatoid arthritis toxicity

Rheumatoid arthritis‐like toxicity is graded up to level 3. It is recommended to suspend ICIs and use prednisone (1 mg/kg/day) for four to six weeks if the symptoms do not improve within two weeks. Other immunosuppressants such as methotrexate, sulfasalazine, or leflunomide can also be used. The initial dosage of methotrexate is 15 mg/week, supplemented with folic acid every day, and titrated to the maximum dose of 25 mg/week. The IL‐6 inhibitor tocilizumab or infliximab can also be used as an anti‐inflammatory treatment for arthritis. The NCCN/ASCO guidelines recommend tocilizumab or infliximab if steroids are contraindicated. Arthritis symptoms can last for more than two years and require the use of immunomodulator drugs, and may also occur with other irAEs (such as Sjogren's syndrome, mumps, inflammatory myositis, vasculitis, rheumatic polymyalgia, lupus erythematosus, and sarcoidosis).

There are reports of severe or refractory arthritis that have been effectively controlled using IL‐1 and‐6 inhibitors, anti‐IL‐17 monoclonal antibodies, anti‐TNFα monoclonal antibodies, anti‐IL‐23 and ‐12 mAb, and Janus kinase inhibitors, and these agents can also shorten the overall medication cycle.^5^

#### Myositis or myalgia

Moderately severe myositis requires discontinuation of ICIs, hospital admission, and immediate treatment with 1–2 mg/kg/day methylprednisolone. For severe and refractory cases, a muscle biopsy should be considered, and continuous monitoring of creatine kinase/aldolase levels, intravenous immunoglobulin treatment, and plasma replacement should be considered until symptoms disappear or steroids are stopped. No immunosuppressive agents (including specific and nonspecific agents) are recommended.

The major guidelines/consensus on myositis do not recommend immunosuppressive therapy, but there are reports of improvement with the use of specific immunosuppressive agents (cytokine antagonists) when combined with myositis and other systemic injuries. Myositis and myocarditis can occur together or separately, and can involve swallowing muscles and respiratory muscles and affect these functions. For severe myositis with extensive muscle involvement, combined with other organs such as the myocardium, or myositis that affects swallowing or respiratory muscles, early treatment with steroid hormones and immune agents can be explored to control muscle inflammation. To reduce muscle damage, after the decline of creatine kinase in the later stage of the disease, the course of high‐dose hormone therapy can be shortened as much as possible, and supplemented with rehabilitation and support therapy to improve the prognosis.

### Neurotoxicity

Regarding neurotoxicity, the guidelines divide it into different types for description and recommendation, which is summarized as follows: (i) Myasthenia gravis: for grade 3–4 toxicity, permanently stop ICIs. Hospitalization is recommended, with initiation of methylprednisolone at 1–2 mg/kg/day. The dose should be adjusted according to the condition, but there are no detailed instructions on how to adjust the dose. Drugs that may increase muscle weakness (such as β‐blockers, magnesium‐containing drugs, quinolones, aminoglycosides, and macrolide antibiotics) should be avoided, and lung function and neurological symptoms monitored. (ii) The classification of Guallain‐Barré syndrome starts from Grade 2. ICIs should be stopped, the patient should be hospitalized or admitted to an intensive care unit, and neurological symptoms and respiratory function should be closely monitored. It is recommended that immunoglobulin, 0.4 g/kg/day, or plasma exchange, be continued for five days. Nonopioid drugs are given to treat pain. Methylprednisolone can also be used experimentally at 2–4 mg/kg/day, and then gradually reduced. There are also recommendations to use methylprednisolone at 1 g/day for five consecutive days, combined with immunoglobulin or plasma exchange. Additionally, there are recommendations to advance immunoglobulin or plasma exchange therapy and gradually increase steroid hormones, although specific amounts are not described in detail. (iii) Aseptic meningitis: for grade 3–4 toxicity, it is recommended to permanently stop ICIs, and the patient should be hospitalized. Before the cerebrospinal fluid (CSF) test results are clear, consider intravenous injection of acyclovir until polymerase chain reaction (PCR) results are obtained, or intravenous administration of acyclovir antiviral therapy. Excluding bacterial and viral infections, close monitoring should be performed without the use of steroid hormones. If moderate to severe symptoms occur, methylprednisolone at 1–2 mg/kg/day can be used to treat encephalitis. If the diagnosis is clear, methylprednisolone 1–2 mg/kg/day should be administered. If the symptoms are serious or oligoclonal bands appear, methylprednisolone at 1 g/day for three to five days should be administered with immunoglobulin at 0.4 g/kg/day for five consecutive days. If the disease progresses or an autoimmune encephalopathy antibody appears, anti‐CD20 monoclonal antibody, such as rituximab, or plasma exchange should be administered. Empirical antiviral treatment (intravenous acyclovir or acyclovir) and antibiotics can be given before CSF test results are known. (iv) Transverse myelitis: permanently stop ICIs, and administer methylprednisolone 2 mg/kg/day, or a dose of methylprednisolone at 1 g/day, continuous for three to five days, immunoglobulin 0.4 g/kg/day for five consecutive days, or plasma exchange, according to the condition. The SITC guidelines divide neurotoxicity into: central injury‐encephalopathy/leukoencephalopathy/reversible posterior encephalopathy syndrome (Pres), and it is recommended to permanently stop the toxicity of grade 3–4 ICIs with an initial dose of steroid hormones at 1–2 mg/kg/day. Prophylactic antibiotics are used for three days without improvement to prepare for dialysis. For peripheral injury consisting of peripheral motor or sensory neuropathy, both are critical grade 3 toxicities, and ICIs should be permanently discontinued. The initial dose of steroid hormones is 1–2 mg/kg/day, and prophylactic antibiotics should also be used.

For the above nervous system irAEs and steroid hormone treatment, it is recommended to administer immunoglobulin combined with plasma exchange, and this can be repeatedly administered according to the course of treatment, that is, once every 21 days, for a total of three to four applications. Among neurological diseases, subacute and chronic inflammatory demyelinating polyradiculoneuritis, myasthenia gravis, encephalitis, aseptic meningitis, and other diseases all result from immunosuppressive therapy other than steroid hormones. Some researchers reported that subacute and chronic inflammatory demyelination and neuritis should be treated with anti‐IL‐1 receptor inhibitors, myasthenia gravis should be treated with anti‐IL‐1 and ‐6 receptor inhibitors and anti‐CD20 monoclonal antibodies, and encephalitis and aseptic meningitis should be treated with anti‐IL‐1 receptor inhibitors and anti‐CD20 monoclonal antibodies.^8^, ^9^, ^10^ Therefore, specific immunosuppressive therapy can be explored in cases of critical neurotoxicity.

### Blood toxicity

#### Anemia

This category includes autoimmune hemolytic anemia and aplastic anemia. ESMO and NCCN/ASCO did not include autoimmune hemolytic anemia. SITC mentioned the disease name, but no treatment opinion was given. Only the CSCO guide issued a more detailed description, with the recommendation to improve the routine blood parameters, reticulocyte count, peripheral blood smear, lactate dehydrogenase, direct and indirect bilirubin, folic acid, vitamin B12, ferritin, serum iron, globin, Coombs direct indirect test, paroxysmal nocturnal hemoglobinuria, and other tests; the effect of drugs, insects, snakes, or hemolytic anemia caused by bites, or bacterial or viral infections should be excluded. It is recommended to permanently discontinue ICIs, administer prednisone 1–2 mg/kg/day, or if necessary, administer immunosuppressive agents such as anti‐CD20 monoclonal antibody rituximab, immunoglobulin, cyclosporine, or mycophenolate mofetil. Red blood cells can be transfused to correct anemia according to guidelines, supplemented with folic acid 1 mg/day.

Regarding aplastic anemia, only the CSCO guidelines include a more detailed description, which states that it is recommended to improve blood tests, reticulocytes, vitamin B12, folic acid, ferritin, serum iron, liver and kidney function, and viruses, and exclude drugs and aplastic anemia caused by radiation, toxins, or viral infections to confirm the diagnosis. It is recommended to suspend ICIs, the patient should be followed‐up closely every day, engage in hematology consultations, and administer hematopoietic growth factor, ATG + cyclosporine, or cyclophosphamide if required. Eltrombopag and supportive treatment can be administered. Blood can be transfused, but all blood products should be irradiated and filtered.

We suggest that instructions should be given on the use of steroid hormones. The current guidelines recommend that the general treatment principles of aplastic anemia should be followed. Whether these treatments are applicable to anemia caused by ICI remains to be further explored in clinical practice.

#### Immune thrombocytopenia

The ESMO and NCCN/ASCO guidelines do not include immune thrombocytopenia. SITC mentions the disease name but provides no treatment recommendations. Only the CSCO guidelines include a more detailed description, consisting of the recommendation to perform routine blood tests, and tests for autoantibodies, detection of platelet antibodies, viruses, or bacteria. At the same time, it is necessary to exclude drugs, other autoimmune diseases, thrombocytopenia caused by viral infections, aplastic anemia, and other diseases to confirm the diagnosis. ICIs should be suspended when toxicity is above grade 3, and close follow‐up and treatment are necessary. If treatment results in a return to toxicity below grade 1, continuation of treatment is acceptable, and oral prednisone 1–2 mg/kg/day can be administered. If there is no remission or deterioration, prednisone can be continued combined with intravenous infusion of immunoglobulin 1 g/kg, and repeatedly used as needed. The use of anti‐CD20 monoclonal antibody rituximab and thrombopoietin receptor agonist eltrombopag should also be considered.^12^

The CSCO guidelines state that “prednisone 1–2 mg/kg/day orally should be administered; if there is no remission or deterioration, continue to use prednisone and combine with intravenous infusion of immunoglobulin 1 g/kg, and repeat use as required”, although we believe that this should be used with caution in clinical practice. For those patients who do not respond to hormones, early consideration should be given to the use of the thrombopoietin receptor agonist eltrombopag, anti‐CD20 monoclonal antibody rituximab, and other drugs.

#### Acquired hemophilia

This disorder is described in more detail in the CSCO guidelines. It is recommended that routine blood parameters, fibrinogen, and prothrombin time (PT) should be obtained. Activated partial thrombin time determination (APTT) should be performed, with any required APTT correction experiments. Coagulation factor quantification and Bethesda coagulation factor inhibitor determination should be performed. Magnetic resonance imaging, computed tomography, or ultrasound should be used to locate, quantify, and continuously monitor bleeding to confirm the diagnosis of hemophilia. For grade 3 or higher toxicity, ICIs should be permanently stopped, and hematology consulted. Clotting factor replacement therapy should be selected according to the expression level of inhibitors by the Bethesda method. The patient should be given 1 mg/kg/day prednisone ± anti‐CD20 monoclonal antibody toximab 1–2 mg/kg/day, cyclophosphamide, and blood transfusion. If the patient's condition continues to deteriorate, cyclosporine or other immunosuppressive agents should be administered.

If immune neutropenia occurs, it is recommended that an intravenous infusion of immunoglobulin 1 g/kg according to the course of treatment should be administered. In addition, there have been reports of eosinophilia and hemophagocytic lymphohistiocytosis associated with ICI therapy.^13^

### Renal toxicity

For grade 3 renal toxicity, ICIs should be permanently discontinued. Creatinine and urinary protein should be monitored every 24 hours, and prednisone or methylprednisolone should be administered at 1–2 mg/kg/day. If the toxicity remains at >2 grades after one week, consider adding azathioprine, cyclophosphamide, cyclosporine, infliximab, or mycophenolate. Emergency dialysis is required for those with life‐threatening toxicity. Dehydration, recent venography, urinary tract infections, drugs, and low or high blood pressure should be excluded, and a renal biopsy should be considered as soon as possible.

It is important to also distinguish different types of kidney injury, such as rapidly progressive glomerulonephritis, immune‐mediated nephritis, immune‐related renal failure, and nephrotic syndrome, and treat them accordingly. For differential diagnosis, bladder involvement should be excluded. The incidence of kidney injury is low, and no additional cases have been reported and accumulated. As for immunosuppressive agents, there are no other recommendations other than the use of infliximab.

### Cardiotoxicity

Grade 3–4 myocardial toxicity requires immediate consultation with a cardiologist to complete the electrocardiogram, markers of myocardial damage (creatine kinase and troponin), and inflammatory markers (erythrocyte sedimentation rate, C‐reactive protein, and white blood cells). Color Doppler ultrasound and/or myocardial enhancement magnetic resonance imaging (MRI) examination with ECG monitoring should be performed, with permanent discontinuation of ICIs. Methylprednisolone shock at 1 g/day for three to five days should be administered. The NCCN/ASCO and CSCO guidelines recommended treatment until cardiac function returns to baseline, with a gradual decrease over four to six weeks. Consider adding ATG and infliximab when hormone therapy does not result in improvement after 24 hours. However, it should be noted that infliximab is related to heart failure, and high‐dose infliximab is contraindicated in patients with moderate to severe heart failure. Myocarditis or pericarditis should be treated with antiviral, immunoglobulin, or dialysis therapy.

We recommend that because myocarditis occurs after receiving ICI treatment during cycles 1–2, clinicians should be vigilant when patients start ICI treatment, the ECG data should be continuously reviewed, in order to detect any changes in the myocardial enzyme spectrum, and strengthen patient education. This is essential for early recognition of myocarditis. A satisfactory outcome was directly related to early detection and treatment.^14^ During carditis, the highest mortality rate occurs for fatal arrhythmia and atrioventricular block. Among these, 36% of patients have complete atrioventricular block, 60% can die, and in 21% the levels of troponin and creatine kinase did not significantly increase in patients with arrhythmia. In such cases, the implantation of cardiac pacemakers and comprehensive specialty cardiology treatment are the most important to improve the prognosis.

In some patients with myocarditis, although myocardial enzymes steadily decrease after pulse steroid therapy, malignant arrhythmia can still occur and lead to death. Myocardial biopsy has also confirmed the existence of uncontrolled inflammation in the myocardium.^15^ For these patients, inhibition drug therapy to gain new specific immunity may be the key to success. We recommend that anti‐IL‐1 receptor inhibitors and anti‐IL‐6 receptor monoclonal antibodies be administered to patients with severe myocarditis on a hormone basis. Anti‐IL‐6 monoclonal antibody is recommended in the treatment of arteritis, and there have also been successful reports of their use in explosive myocarditis secondary to adult Still disease,^16^ and its safety has also been confirmed.

In addition, there are reports in the literature of using the anti‐CD52 monoclonal antibody allemtuzumab to clear peripheral blood T cells to successfully cure refractory myocarditis,^17^ the use of the CTLA‐4 agonist abataxicept (500 mg every two weeks, for a total of five doses) competitively combined with CD80/CD86 to inhibit the T cell costimulatory signaling pathway for refractory myocarditis,^18^ and treatment with antithymocyte globulin ATG (500 mg/day, for 5 days).^15^ However, the safety of these drugs is the main factor restricting their use. There is an increased risk of secondary infection accompanying their use, and a common cause of death in severe myocarditis is when secondary infection occurs after immunosuppression.

### Ocular toxicity

Posterior uveitis or total uveitis is a third degree toxicity. According to the severity of the onset, there is an early benefit to treatment with ICIs and response to glucocorticoid treatment, a small number of grade 3 toxicity patients will be able to resume ICI treatment. For fourth degree toxicities, an ophthalmologist should be consulted before starting hormone therapy, and local or systemic glucocorticoid therapy should be administered as recommended, but ICIs should be permanently discontinued.

Scleritis constitutes grade 3–4 toxicity, and ICIs should be permanently discontinued. According to the severity of the disease, there is an early benefit to treatment with ICIs and the response to glucocorticoid treatment, a small number of grade 3 toxicity patients will be able to resume ICI treatment. An ophthalmologist should be consulted if there is grade 3–4 toxicity before initiating hormone therapy, and local or systemic glucocorticoid therapy should be used as recommended.

Orbititis, blepharitis, optic nerve edema, ulcerative conjunctivitis, and macular degeneration can also be caused by ICI treatment. In addition, there are reports of Vogt‐Koyanagi‐Harada syndrome that has developed with serous retinal detachment.^19^ Other reported ocular abnormalities include macular edema, retinopathy, ocular myositis, orbital inflammation, corneal perforation, and intraocular inflammation. There are few reports on the application of immunosuppressive agents other than steroid hormones for these conditions. However, from the perspective of pathogenesis and organ characteristics, the application of immunosuppressants is still promising.

Based on the guidance of the five irAE‐related guidelines/consensus, we found that due to a lack of large‐scale clinical research and experience, this part of the guidelines on critical/refractory irAEs was not systematic, comprehensive, or detailed, and lacked supportive evidence. Some of the above‐mentioned supplements or suggestions beyond the guidelines are still not perfect, but they can be used as directions for future exploration in irAE management.

## Preliminary discussion on the application of drugs other than steroid hormones for the treatment of irAEs

At present, the treatment of irAEs is still based on steroid hormones. The treatment of refractory irAEs presents the greatest difficulty, and it has been noted that new, rare, and sometimes fatal irAEs are still emerging. For this type of unique disease with a clear etiology and mechanism, how to quickly suppress the immune treatment, control the side effects of the immunosuppressant itself, and optimize choices for organ‐specific treatment may be the key to improving the success rate in the future. Therefore, it is necessary to develop new biological agents and drugs targeting cytokines for effective treatment. Table 1 shows some of the cytokine‐targeted immunosuppressants involved in the guide, and their conventional methods of use.

**Table 1:** Recommended cytokine‐targeted immunosuppressive agents

	Properties	Generic name	Usage and dosage
1	IL‐6 receptor antagonist	Tocilizumab	8 mg/kg, intravenous, once a month, or 162 mg, subcutaneous injection, once a week
2	Immunoglobulin	Immunoglubin	400 mg/kg/day, intravenous for five days
3	Anti‐CD20 antibody	Rituximab	1 g, once every 14 days, for two cycles, or 375 mg/m2, once a week for four cycles
		Ofatumumab	300 mg on day 1, 1000 mg on day 2
		Obinutuzumab	1000 mg on day 1
		Ocrelizumab	300 mg on day 1, 300 mg on day 4
4	Anti‐TNFα antibody	Infliximab	5 mg/kg, once every two weeks
		Adalimimab	40 mg, once every two weeks
		Golimumab	50 mg, once a month
		Certolizumab	400 mg, once a month
		Etanercept	50 mg, once a week
5	Anti‐integrin a4 antibody	Natalizumab	300 mg, once a month
		Vedolizumab	300 mg, once a month
6	Thrombopoietin receptor agonist	Eltrombopag	50 mg/day, orally
7	Antithymocyte globulin	ATG	500 mg/day, days 1–5
8	Janus kinase inhibitor	Tofacitinib	2 mg, b.i.d., orally

The use of some cytokine‐targeted immunosuppressants in irAEs has been recommended in the guidelines. For example, the use of anti‐TNFα monoclonal antibodies is recommended for myocarditis and colitis, and it is necessary to further clarify its scope of application and side effects. Antagonists against IL‐6 exhibit a satisfactory effect when administered in response to the cytokine release syndrome caused by CAR‐T cells, and they have also shown certain effects in the treatment of critical and refractory irAEs.^20^ Immune injury results due to the cytokines that are continuously secreted during the acute inflammation period, and they are also responsible for the subsequent immune response cascade, especially that consisting of IL‐1, IL‐6, and TNFα. Blocking these cytokines will reduce their effect on helper T cells, B cells, and will naturally stimulate the effect of killer cells, macrophages, plasma cells, hematopoietic stem cells, and cause endothelial activation. This may be more effective than the anti‐TNFα strategy that is classically advocated.

Because of the tumor‐promoting and metastasis‐promoting activities of IL‐6 and IL‐1, it is also possible to achieve additional antitumor advantages by preferentially using anti‐IL‐6 or −1 monoclonal antibodies to achieve a “shutdown” strategy. In cases of encephalitis induced by ICIs, anti‐IL‐1 can be administered as a useful adjuvant therapy, in which the inflammatory response is mainly driven by an increase in IL‐1. Anti‐B cell antibodies may contribute to neurological or hematological complications of ICI and connective tissue diseases induced by ICI, severe Stevens‐Johnson syndrome, and irAEs associated with vasculitis. In addition, IL‐12/23 inhibitors can suppress the acute inflammatory phase by weakening the positive stimulation of IL‐23 on TNFα secretion, and can be utilized in irAE cases where the efficacy of anti‐TNFα drugs is low. In the case of refractory psoriasis‐like reactions after failure of anti‐IL‐6 or anti‐TNFα treatments, an anti‐IL‐17 strategy may be an excellent choice.

To further explore the treatment of critical and refractory irAEs, drugs should be selected after hormone resistance occurs. It should also be determined whether pharmaceutical agents can be linked to cytokine‐targeted immunosuppressive therapy at the early stage of the disease, so as to suppress the pathological damage that rapidly progresses at that time. The period of high immune suppression in the later period should be minimized to reduce the risk of infection, and finally achieve a successful treatment.^5^

## Conclusions

Steroid hormones can treat most irAEs, especially minimal irAEs, but there are refractory irAEs that are partially steroid‐resistant or cannot be treated with the use of simple steroid hormones. These require the application of other immunosuppressants, especially the specific application of immunosuppressants. Very large doses of steroid hormones are only recommended in a few cases. Repeated use of large doses of hormones should be avoided, except for cases of immune thrombocytopenia. During the treatment of critical illness, the observation methods to determine clinical efficacy include the evaluation of symptoms, signs, and functions, laboratory examinations, and imaging studies. Other interventions should also be performed. For a specific irAE, specialized supportive treatment, including surgical intervention, is important, but it must be considered in combination with the characteristics of patients with advanced tumors and the characteristics of the course of the irAE. During the management of irAEs, it is necessary to note the behavior of malignant tumors.
